# Teaching Pharmacy Undergraduate Students Inhaler Device Technique and Exploring Factors Affecting Maintenance of Technique

**DOI:** 10.1155/2018/1597217

**Published:** 2018-07-03

**Authors:** Mariam Toumas-Shehata, Mark Henricks, Ludmila Ovchinikova, Lorraine Smith, Sinthia Bosnic-Anticevich

**Affiliations:** ^1^Woolcock Institute of Medical Research, 431 Glebe Point Road, Glebe, NSW 2037, Australia; ^2^Utrecht University, P.O. Box 80.115, 3508 TC Utrecht, Netherlands; ^3^The University of Sydney, A15–Pharmacy and Bank Building, Sydney, NSW 2006, Australia

## Abstract

**Aim:**

To determine the effect of inhaler device technique education on the maintenance of the inhaler device technique of undergraduate pharmacy students over time and to determine the factors, if any, related to inhaler device technique maintenance in the academic setting.

**Methods:**

This study took the form of a prospective, unpaired samples repeated measures design. Participants had their inhaler technique assessed at baseline. Participants were then shown how to use an inhaler as a group. Participants then worked in pairs and took turns teaching and assessing each other on the correct inhaler technique using the 9-step checklist. Immediately following the delivery of the intervention, participants had their inhaler technique reassessed. All participants were then trained to mastery through individualised training. Twelve months following the collection of baseline data, all participants had their inhaler technique assessed and completed two questionnaires.

**Results:**

Following the delivery of the intervention, there was a significant increase in the proportion of participants with the correct inhaler technique when compared to baseline (11% to 61%, resp.). There was a significant reduction in the proportion of participants demonstrating the correct technique 12 months following training (28%). The strongest determinant of inhaler technique maintenance is experience with showing patients how to use their inhalers.

**Conclusion:**

Repeated training is essential to ensure that the technique is mastered and maintained, and consolidation of skills through exposure to train others may be most effective.

## 1. Introduction

The optimal use of medications is generally considered an integral part of chronic disease management and is an obvious focus for many pharmacy interventions [1]. In the case of asthma management, optimal use of asthma medications is complicated by the fact that asthma medicines are delivered via inhalation and therefore, patients are required to not only understand when to use their medications but also to develop the skills to physically use their asthma inhaler devices correctly [2].

Much research has focused on the use of inhaler devices and universally confirmed that a majority of patients are not able to use their inhalers correctly [3–9]. This tends to be the case regardless of which inhaler devices are used or how long a patient has been using them for [3–9].

The implications of incorrect inhaler device use are significant. Research has shown that the incorrect inhaler device technique can lead to less than half the expected dose being delivered to the airways [10], while improvements in the inhaler device technique have been shown to lead to improved lung function, asthma control, and asthma quality of life [7, 11, 12].

Inhaler device educational interventions delivered in the community pharmacy setting have been shown to be effective in improving the inhaler technique [6, 13–15]. However, data indicate that many patients do not receive education in an effective manner, that is, through the use of a placebo inhaler and involving a physical demonstration of correct use by the health care professional [1, 6, 16].

When it comes to the inhaler technique and health care professionals, it is clear that part of the problem with incorrect use amongst patients could be associated with the low level of awareness of the correct inhaler technique by health care professionals [17]. Studies indicate that up to 85% of health care professionals are not able to use the inhaler devices correctly [18–21].

Understanding and being able to demonstrate correct device handling in asthma is a core activity of pharmacists, and therefore, in pharmacy schools, where pharmacy students' preliminary knowledge is obtained, inhaler technique education is initiated. Different methodologies have been investigated to optimize inhaler technique demonstration skills amongst pharmacy students; however, none have been able to achieve perfect technique in all students [22–27]. The reasons for this have not been explored; however, we can hypothesize that it may be due to the nature of education provided to students and the need to revisit inhaler technique education regularly. Both of these have been shown to be important in the training of patients in device mastery and maintenance [1, 2].

Given the importance of the inhaler device technique in the management of asthma and need to provide effective and efficient modes of training in medication management, which in the case of respiratory management involving the correct use of inhalers, the effectiveness of teaching students this skill during their university training became a relevant pedagogical question.

The aim of this study was to determine the effect of inhaler device technique education on the maintenance of the inhaler device technique of undergraduate pharmacy students over time and to determine the factors, if any, related to inhaler device technique maintenance in the academic setting. In order to achieve this aim, students were followed across 2 academic years. The Turbuhaler® (TH) was chosen as the inhaler in this study despite there being several respiratory devices currently available on the market as it is one of the long-standing, commonly prescribed devices which play an important role in preventer drug delivery [1].

## 2. Materials and Methods

This study took the form of a prospective, unpaired samples repeated measures design in which data were collected on 2 occasions, 12 months apart. The research reported in this manuscript was approved by the University of Sydney Human Ethics Committee.

### 2.1. Participant Recruitment

This study was conducted within one of the core pharmacy practice courses/units of study within the early stages of a pharmacy curriculum (Year 2). All students enrolled in the particular unit of study were invited to participate in this study. Participation was dependent on the student giving written informed consent.

The study was conducted within the 3-week respiratory block, which consisted of 7 lectures and one 2-hour tutorial/workshop. All students were required to attend the tutorial, which was conducted at the end of the 7 respiratory lecture series. The focus of the tutorial was the management of asthma, which included hands on training in inhalers.

At the start of the tutorial, each participant was given a placebo inhaler TH and an information leaflet explaining the use of the device (package insert product information). Each participant was given 10 minutes to practice using the inhaler, referring only to the written resources provided. Participants were not instructed on how or what to do with the placebo device, nor how to use the written information provided. Participants utilized their own learning techniques.

### 2.2. Baseline Assessment/Pretraining (*T* = *t*_1*a*_)

After 10 minutes of self-training, all participants had their inhaler technique assessed by one of 3 assessors who had previously undergone assessor training. Participants were asked to demonstrate the correct use of an inhaler using the placebo device and were assessed using the 9-step checklist [6]. The correct inhaler technique corresponded to a score of 9/9.

Participants were also asked to respond to two questions which aimed at identifying participants' exposure to/experiences with demonstrating or using an inhaler TH, namely, (i) Do you need to use a TH respiratory device in your daily life (e.g., because of a medical condition such as asthma)? (ii) Have you shown anyone how to use their respiratory device (e.g., at your place of work?). Each of these questions related to a different type of exposure/experience to inhaler use and was not mutually exclusive. Dichotomous responses of “Yes” or “No” were recorded for each question.

### 2.3. Peer Training (*T* = *t*_1*b*_)

Participants were then shown how to use an inhaler as a group, by watching a step-by-step demonstration completed by the tutor using a placebo inhaler (an inhaler technique expert). Participants then worked in pairs and took turns teaching and assessing each other on the correct inhaler technique using the 9-step checklist. Immediately following the delivery of the intervention, participants had their inhaler technique assessed by an assessor. Participants were asked to demonstrate the correct use of an inhaler using the placebo device and were assessed using the 9-step checklist [6].

### 2.4. Individualised Training (*T* = *t*_1*c*_)

Following this, all participants were trained to mastery on an individual base, by the tutor who performed a step-by-step demonstration with a placebo inhaler that specifically addressed the errors made by individual students. This was repeated on an individualised basis until each student was able to use a TH correctly.

### 2.5. Follow-Up (*T* = *t*_2_)

Twelve months following the collection of baseline data, all potential participants were approached by a researcher who was blinded to all baseline data collection and were asked to participate in a follow-up of the inhaler technique. Students who had completed the devices training workshop twelve months prior were eligible to participate. Potential participants were informed about the aims of the follow-up phase of the study, and written informed consent was again obtained from each participant.

#### 2.5.1. Self-Efficacy for Asthma Device Use and Inhaler Use

Following the signing of informed consent, participants were asked to complete two questionnaires.

**Figure 1:**
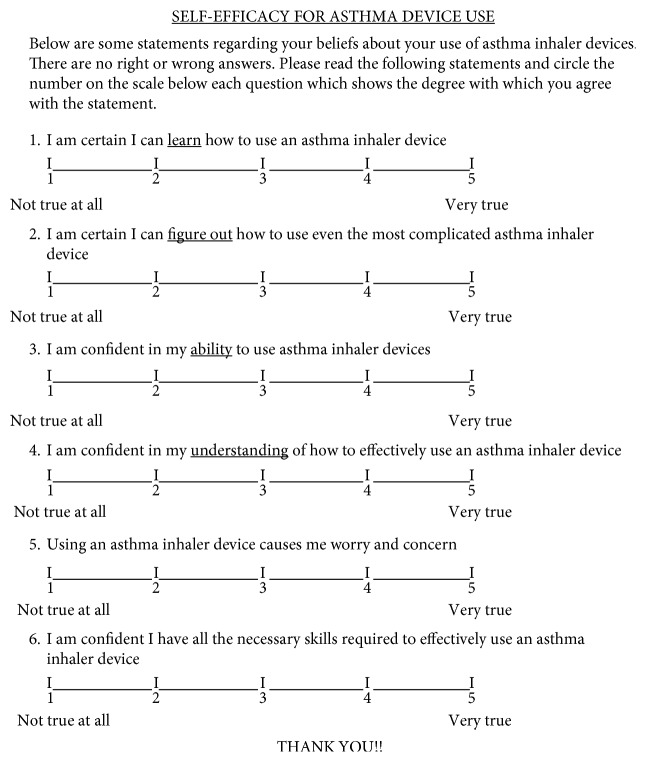
Self-Efficacy for Asthma Device Use questionnaire.

The “Self-Efficacy for Asthma Device Use” questionnaire was developed to evaluate the participant's self-efficacy with inhaler devices and was based on established construct of self-efficacy for learning a skill [21]. It consisted of 6 items which tapped into students' perceptions of confidence in their ability to learn and to understand how to use an asthma inhaler device, to use a device, to have the skills required to use a device, and to be untroubled by anxiety when using a device and their capacity for device use regardless of the difficulty involved (Figure 1). Each of these items was scored on a 5-point Likert Scale, and a total score out of 30 was calculated for each participant.

**Figure 2:**
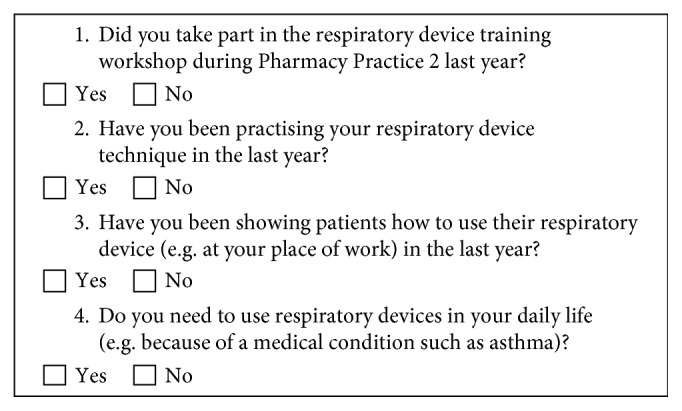
“Inhaler Use” questionnaire.

The “Inhaler Use” questionnaire consisted of 4 questions and aimed at identifying participants' exposure to/experiences with demonstrating or using an inhaler TH since they had completed the devices training workshop twelve months prior (Figure 2). Each of these questions related to a different type of exposure/experience to inhaler use and was not mutually exclusive. Dichotomous responses of “Yes” or “No” were recorded for each question.

#### 2.5.2. Inhaler Technique Assessment

All participants had their inhaler technique assessed by one of 3 assessors who had previously undergone assessor training. Participants were asked to demonstrate the correct use of an inhaler using the placebo device and were assessed using the 9-step checklist [6]. The correct inhaler technique corresponded to a score of 9/9.

### 2.6. Data Analysis

Data relating to the inhaler technique score were collected at pretraining (*T* = *t*_1*a*_), peer training (*T* = *t*_1*b*_), individualised training (*T* = *t*_1*c*_), and follow-up (*T* = *t*_*2*_).

In order to determine the effect of inhaler device technique education on the maintenance of the inhaler device technique of pharmacy students, over time, independent samples repeated measures analysis was undertaken to compare the mean inhaler technique score at *t*_1*a*_, *t*_1*b*_, *t*_1*c*_, and *t*_2_. Due to the deidentification of original data collected, it was not possible to match data for individuals, hence the need to perform independent samples analysis.

In order to determine the factors related to correct inhaler device technique maintenance, a multiple regression analysis with backward elimination was used to determine the relationship between inhaler technique maintenance (a dependent variable) and the independent variables of self-efficacy with the inhaler devices score and inhaler use (a practice of the inhaler technique over the last 12 months, showing patients how to use and need to use in daily life).

## 3. Results and Discussion

### 3.1. Results

All 236 second-year pharmacy students invited to participate in the study agreed to do so. At *T*  = *t*_1*a*_, 11% of participants demonstrated correct technique. Following the delivery of peer training at *T* = *t*_1*b*_, there was a significant increase in the proportion of participants with the correct inhaler technique when compared to baseline (from 11% to 61%, resp.; *n*=236, *p*=0.04). When the inhaler technique was expressed as a mean score out of 9 (as per the 9-item checklist), there was a significant improvement in the mean TH score following peer training (*T* = *t*_1*b*_) (6.6 ± 1.4 at *T* = *t*_1*a*_ compared with 8.3 ± 1.1 at *T* = *t*_1*b*_; *n*=236, *p* < 0.001). All students were trained to mastery by the end of the tutorial (*T* = *t*_1*c*_).

With regard to the previous inhaler use, 41 out of 236 (17%) participants had previously used a TH. There was no significant difference in the proportion of participants with the correct technique at baseline (*p*=0.40) or after peer training (*p*=0.30) for participants who had previously used a TH compared with those who had never used it.

### 3.2. Results at Follow-Up (*T* = *t*_2_)

Of the 280 students enrolled in Year 3 of undergraduate Bachelor of Pharmacy degree in 2008, 236 were eligible (based on enrolment at baseline) and 200 agreed to participate (a response rate of 85% (200/236).

The mean (±SD) score for Self-Efficacy for Asthma Device Use was 23.82 ± 3.97, ranging from 12 to 30, while results relating to inhaler use since completing the inhaler device workshop twelve months prior indicate that 13% (26/200) of participants had practiced using an inhaler (TH), 23% (45/200) had shown patients how to use an inhaler (TH), and 8% (15/200) needed to use an inhaler device in their daily life because of a medical condition.

**Figure 3:**
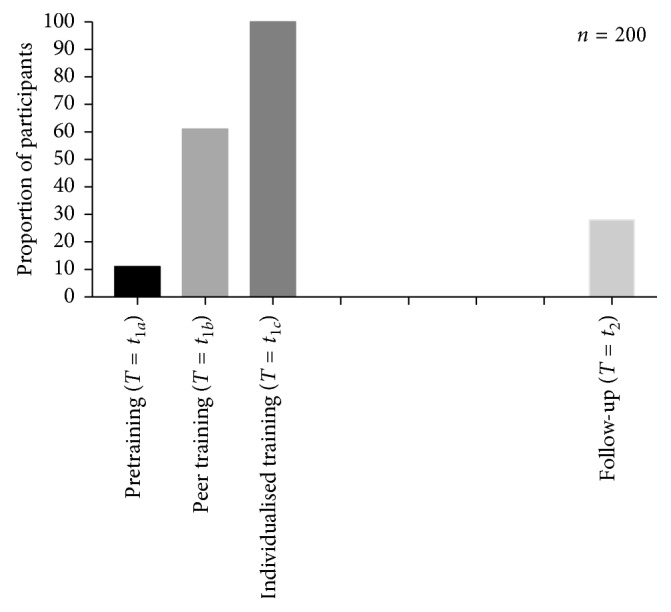
Proportion of participants demonstrating the correct technique during different phases of training and over time.

A one-way repeated measures ANOVA was conducted to compare inhaler technique scores of the 200 participants for whom data across all time points was available, that is, pretraining (*T* = *t*_1*a*_), peer training (*T* = *t*_1*b*_), individualised training (*T* = *t*_1*c*_), and follow-up (*T* = *t*_2_) (Figure 3). The means and standard deviations are presented in Table 1. There was a significant decrease in the mean inhaler technique score at follow-up (*T* = *t*_2_) compared to posttraining (*T* = *t*_1*c*_) (Wilk's lambda = 0.386, *F* (2, 28) = 174.29, *p* < 0.001). Twenty-eight percent of participants were able to demonstrate the correct inhaler technique, that is, achieved a score of 9/9, at follow-up (*T* = *t*_2_) compared with 61% postpeer training (*T* = *t*_1*b*_), and 100% postindividualised training (*T* = *t*_1*c*_), indicating a significant reduction in the proportion of participants demonstrating the correct technique 12 months following training.

Participants who had shown patients how to use their inhaler device or had practiced using an inhaler had significantly higher Self-Efficacy for Asthma Devices Score and Inhaler technique score at follow-up compared to those who had not (Levene's test for equality of variances *p* < 0.05, *n*=200). However, participants who had been using an inhaler device in their daily life had significantly higher Self-Efficacy for Asthma Devices Score but not Inhaler technique score compared to those who had not used an inhaler in their daily life (Levene's test for equality of variances *p* < 0.05, *n*=200).

With regard to the factors determining inhaler device technique maintenance, multiple regression analysis with backward elimination indicated the strongest correlations were between (I) Self-Efficacy for Asthma Devices Score and experience with showing patients and (II) Inhaler technique score and experience with showing patients (Table 2). The final model indicates that the strongest determinant of inhaler technique maintenance is experience with showing patients how to use their inhalers (Table 3).

### 3.3. Discussion

Pharmacists obtain inhaler technique training from various sources, such as package inserts and training from pharmaceutical representatives [6, 22]. However, for many pharmacists, their first inhaler educational experience is in the pharmacy school [23], suggesting that this is the place where their preliminary knowledge is obtained. Students should therefore be able to demonstrate a correct inhaler technique in order to train others. This research shows that even when students are trained how to use inhaler devices, this skill is not maintained. It is only those students who practice using inhalers or are actively showing others how to use inhalers are more likely to maintain this skill. This has significant implications for the training of the inhaler technique to students worldwide as these findings serve as a cornerstone for the development of better future education and training strategies in this domain.

This study served as a proof of concept study; therefore, in designing the study, it was important to ensure that one inhaler was chosen for experimentation. The TH was chosen as the inhaler in this study despite there being several respiratory devices currently available on the market as it is one of the long-standing, commonly prescribed devices which play an important role in preventer drug delivery [1]. While there are other new devices currently available, not all of them are available globally. Further, while the optimal use of different inhalers necessitates different steps, much research over many years has shown that the inhaler technique is a generic problem which continues to be problematic, despite the availability of newer devices [28]. Therefore, it is considered that the results from this research add to our fundamental understanding of the mastering and maintenance of the inhaler technique more broadly. Future research should now explore these findings with regard to other devices as well as with practising pharmacists and other health care providers, especially as this study suggests that if the inhaler technique is not being shown to patients, health care provider mastery is not maintained. This can potentially become a vicious cycle of failure to train and failure to continue to have the ability to train. Severity of errors was also not distinguished in this study as future health care professionals should have the skills to train every step when it comes to educating patients.

Prior to baseline assessment, students were given written information and a placebo inhaler device. This part of the study was aimed at mimicking the real-life scenario in which pharmacists would have access to information about the device technique through the product information leaflet and would be required to study this independently. Only a small proportion of students (11%) were able to use the TH correctly after reading the written information provided. This is consistent with the literature that indicates that when it comes to device technique training, verbal instruction and written information are not effective in improving the technique [7]. Physical demonstration is the most effective method of educating both students and patients on the correct inhaler technique [6, 7].

Of the 200 participants at follow-up (*T* = *t*_2_), 28% were able to demonstrate the correct inhaler technique, a significant drop from the 100% following individualised training (*T* = *t*_1*c*_). This proof of concept study demonstrated that despite training to mastery, the ability of pharmacy students drops significantly and reeducation is required. What this study fails to answer is at what point does mastery drop-off and hence, at what stage should additional training be provided. Future research should aim at articulating this detail of training, keeping in mind that when it comes to training health care provider students, university curricula often determine the content of education; therefore, the scheduling of further inhaler technique training would need to be sympathetic to the overall training curriculum.

This finding is consistent with other studies looking at maintenance of the technique following the training of fully qualified health care professionals (including physicians and pharmacists) [24, 25]. In 1996, Rebuck and Dzyngel [24] educated postgraduate physicians on the correct inhaler technique in one brief structured educational intervention with hands-on education. Results revealed a significant improvement in the inhaler technique scores following this training. However, 8 months postintervention, 59% of the physicians were found to demonstrate poor technique. This was also further highlighted by Resnick and Gold [25] who delivered education on the correct inhaler technique to paediatric house-staff physicians through a single-inhaler training session. Technique was found to deteriorate 2 months following the education. This highlights the importance of periodic retraining on the correct inhaler technique in order to maintain effective patient educators [24] and suggests that inhaler technique competency needs to be assessed and corrected at regular intervals after education to help maintain the optimal technique.

The “Self-Efficacy for Asthma Device Use” questionnaire was developed to evaluate the participant's self-efficacy with inhaler devices and was based on established construct of self-efficacy for learning a skill [21] with the purpose of gaining further insights into inhaler technique maintenance. Self-efficacy is defined as a person's belief in their ability to succeed in specific situations or accomplish a task. The person's sense of self-efficacy plays a major role in how they approach tasks and challenges. The strongest determinant of inhaler technique maintenance was found to be experienced with showing patients how to use their inhalers. Analysis indicated that those participants who had shown patients how to use their inhaler devices had significantly higher Self-Efficacy for Asthma Devices Score and Inhaler technique score compared to those who had not. Showing patients how to use their inhalers has been of great benefit in helping students to maintain their technique. In a study by Basheti et al. [26], pharmacy students were asked to identify different barriers to learning the correct inhaler technique. Students identified one of the biggest barriers was the lack of engagement with patients who use these devices [26]. Perhaps, students' lack of engagement with real patients who use these inhalers may contribute to the decline in inhaler device technique maintenance, and perhaps, engaging pharmacy students with periodic, regular, real patient counselling, or some sort of simulation-based leaning [27] may help with the maintenance of the correct technique.

Participants who had been using an inhaler device in their daily life had significantly higher Self-Efficacy for Asthma Devices Score but not Inhaler technique score at follow-up compared to those who had not. Although this appears somewhat inconsistent as one presumes that they would follow the correct technique more than the general population because they know better, studies have shown that this is not always the case and in fact health care professionals do not always practice what they “preach” [29]. One study has shown that it was clear that the standard management procedures and instructions concerning basic health-preserving behaviour were far from being universally accepted and followed by health workers themselves. This was the case even for common diseases and health issues for which specific training had been given [29]. Possible reasons for this include habit, lack of motivation, and they are behaving as a patient rather than a health care professional in the management of their own illness.

In reviewing these results, it is important to consider the limitations of this research. The lack of data matching with students meant it was not possible to match baseline and follow-up cases. This may have had an impact on the strength of the relationships determined, that is, the results of this study may be overly conservative to the real-life scenario. Other limitations include the use of only one pharmacy student cohort from one university. However, no major differences can be found in the related educational methodologies used in other schools of pharmacy that would suggest a need to limit the generalization of the findings of this study. In training the students in the inhaler technique, evidence educational interventions were used, which are hopefully also applied in other academic settings.

## 4. Conclusion

Training pharmacy students in the correct use of inhaler devices is a transient skill, and this study confirms that training health care provider students (in this case pharmacy students) in a one-off session is not sufficient to ensure maintenance of the correct technique over time. This may be due to inadequate practice with real patients or lack of retraining students regularly.

The practical implications of this research are clear: repeated training is essential to ensuring technique is mastered and maintained, and consolidation of skills through exposure to train others may be most effective.

## Figures and Tables

**Table 1:** Proportion of participants with the correct inhaler technique (i.e., achieving a score of 9/9) at baseline, peer training, individualised training, and at follow-up.

	Baseline assessment (*T* = *t*_1*a*_)	Peer training (*T* = *t*_1*b*_)	Individualised training (*T* = *t*_1*c*_)	Follow-up (*T* = *t*_2_)	*P*
Proportion of participants with correct technique (%)	11 % (260/236)	61 % (144/236)	100% (236/236)	28% (56/200)	0.004^a^
Mean inhaler technique score (+/−SD)	6.6 (+/−1.4)	8.3 (+/−1.1)	9	6.9 (+/−1.9)	≤0.001^b^

^a^Chi-square test for independence, *χ*^*2*^ (1, *n*=200) = 8.56, *p*=0.004, phi = 0.19; ^b^one-way repeated measures ANOVA (Wilk's lambda = 0.386, *F* (2, 28) = 174.29, *p* ≤ 0.001, *n*=200).

**Table 2:** Correlations between total Self-Efficacy for Asthma Devices Score, inhaler device score at follow-up, and inhaler use (personal practice, showing patients, and daily use).

		Total SE score	Total IDT score	Experience with practicing	Experience with showing patients	Experience by daily use
Total SE score	Pearson correlation	1	0.217^a^	0.289^a^	0.318^a^	0.229^a^
	Significance (2-tailed)		0.001	0.000	0.000	0.001

Total IDT score	Pearson correlation		1	0.184^a^	0.204^a^	0.090
	Significance (2-tailed)			0.006	0.002	0.182

Experience with practicing	Pearson correlation			1	0.379^a^	0.364^a^
	Significance (2-tailed)				0.000	0.000

Experience with showing patients	Pearson correlation				1	0.234^a^
	Significance (2-tailed)					0.000

Experience by daily use	Pearson correlation					1
	Significance (2-tailed)					

^a^Correlation is significant at the 0.01 level (2-tailed).

**Table 3:** Outcomes of multiple regression analysis with adjusted *R* square = 0.063, *F* = 4.295, *p* < 0.0005, *n*=200.

Predictor variable	Beta	*p*
Self-efficacy score	0.115	0.125

Inhaler use		
Shown patients	0.157	0.045
Practiced	0.128	0.136
Daily use	−0.043	0.592

## Data Availability

The data used to support the findings of this study were provided by the University of Sydney under license and so cannot be made freely available. Access to these data will be considered by the authors upon request.
